# Integrated Bioinformatics and Validation Reveal IL1B and Its Related Molecules as Potential Biomarkers in Chronic Spontaneous Urticaria

**DOI:** 10.3389/fimmu.2022.850993

**Published:** 2022-03-18

**Authors:** Shixiong Peng, Teng Zhang, Sisi Zhang, Qian Tang, Yang Yan, Hao Feng

**Affiliations:** ^1^ Department of Dermatology, The First Affiliated Hospital of Hunan Normal University/Hunan Provincial People’s Hospital, Changsha, China; ^2^ Department of Dermatology, Chinese Traditional Hospital of Changsha, Changsha, China; ^3^ Nursing Department, Hunan Provincial People’s Hospital/The First Affiliated Hospital of Hunan Normal University, Changsha, China

**Keywords:** chronic spontaneous urticaria (CSU), pyroptosis-related genes, IL1B, bioinformatics, inflammation, immunology

## Abstract

**Background:**

The etiopathogenesis of chronic spontaneous urticaria (CSU) has not been fully understood, and there has been extensive interest in the interaction between inflammatory dermatosis and pyroptosis. This study intends to investigate the molecular mechanism of pyroptosis-related genes in CSU *via* bioinformatic ways, aiming at identifying the potential key biomarker.

**Methods:**

GSE72540, the RNA expression profile dataset of CSU, was utilized as the training set, and GSE57178 as the validation set. Differently expressed pyroptosis-related genes (DEPRGs), GO, KEGG, and DO analyses were performed. The hub genes were explored by the protein–protein interaction analysis. Moreover, CIBERSORT was employed for estimating immune cell types and proportions. Then, we constructed a DEmRNA–miRNA–DElncRNA ceRNA network and a drug–gene interaction network. Finally, ELISA was used for gene expression analysis.

**Results:**

We recognized 17 DEPRGs, whose enrichment analyses showed that they were mostly enriched in inflammatory response and immunomodulation. Moreover, 5 hub genes (IL1B, TNF, and IRF1 are upregulated, HMGB1 and P2RX7 are downregulated) were identified *via* the PPI network and verified by a validation set. Then immune infiltration analysis displayed that compared with normal tissue, CSU owned a significantly higher proportion of mast cells activated, but a lower proportion of T cells CD4 naive and so on. Furthermore, IL1B was statistically and positively associated with mast cells activated in CSU, and SNHG3, the upstream factor of IL1B in the ceRNA we constructed, also related with mast cells in CSU. Further analysis exhibited that the protein subcellular localization of IL1B was extracellular, according with its intercellular regulation role; IL1B was significantly correlated with key immune checkpoints; and the NOD-like receptor signaling pathway was the mainly involved pathway of IL1B based on the couple databases. What is more, the result of ELISA of CSU patients was the same as the above analyses about IL1B. In addition, the drug–gene interaction network contained 15 potential therapeutic drugs targeting IL1B, and molecular docking might make this relationship viable.

**Conclusion:**

IL1B and its related molecules might play a key role in the development of CSU and could be potential biomarkers in CSU.

## Introduction

Chronic spontaneous urticaria (CSU) is one of chronic inflammatory dermatosis, which is delineated as, for recognized or unrecognized causes, angioedema, wheal, or both occurring spontaneously for more than 6 weeks (1). The CSU’s prevalence is approximately 1% of the population (lifetime prevalence = 1.4%; point prevalence = 0.7%) (2). Moreover, CSU will get the increase of risk for comorbid autoimmune diseases like autoimmune thyroid disease (3). The frequently recurring symptoms, pruritus, urticaria, and angioedema, severely affect patients’ performance at school and work and impair their quality of life, which brings much encumbrance to both their households and society (4). Unlike acute urticaria, which is usually caused by an identifiable agent like an allergic reaction to a drug or other, the cause and pathogenesis of CSU are complex and remain largely unclear (5). Consequently, it is of great significance for individualized and effective treatment to reveal the pathogenesis and recognize key biomarkers of CSU.

The CSU’s etiopathogenesis has not been totally uncovered, but the existing studies suggest that maladjustment of inflammatory cells (such as mast cells and basophils) is the potentially core contributor (6). It is well known that a series of intracellular signaling cascades result in mast cell activation, after IgE bonds to the high-affinity IgE receptor. The activated mast cell releases proteases, histamines, and cytokines with the generation of platelet-activating mediators and other arachidonic acid metabolites (leukotrienes C4, D4, and E4 and prostaglandin D2). These cytokines give rise to vascular permeability and increased vasodilation, ensuing interstitial edema and sensory nerve stimulation and causing the obvious itchiness, redness, and swelling (7, 8). Besides, some CSU patients could show signs of activation of the coagulation/fibrinolytic system, such as significant elevation of serum factors like D-dimer, sICAM-1, and sVCAM-1 (9, 10). In this setting, some treatment strategies were developed, such as antihistamine, biological agent, and immunosuppressant. The first-line symptomatic treatment for CSU is largely depending on modern 2nd-generation H1 antihistamines, but standard-dosed antihistamines are ineffective in about 40% of the patients (11). Omalizumab, a humanized anti-IgE antibody, is the first licensed biological treatment by the Food and Drug Administration (FDA) for patients with CSU refractory to H1 antihistamines. However, relapse rates following the withdrawal of omalizumab are high (12). Ciclosporin is recommended for combination treatment in patients with severe disease refractory. Nevertheless, it is a problem which cannot be ignored that long-term use of ciclosporin leads to serious side effects (13). It can thus be seen that complete control of symptoms in the majority of patients remains a worldwide challenge. Consequently, further and fuller exploring the inflammatory reaction pathogenesis of CSU is scientifically significant to the clinical therapy.

There has been extensive interest in the interaction between inflammatory reaction and pyroptosis. The pyroptosis is denoted as inflammasome-dependent cell death (14). It was found that it makes a critical difference in the development of numerous inflammatory skin diseases. A research reported that Mdivi-1 significantly suppressed the pyroptotic cell death of keratinocytes and inhibited NLRP3 inflammasome activation to play a protective role in atopic dermatitis (15). Deng et al. indicated that cycloastragenol could inhibit the liberation of inflammatory mediators and macrophage infiltration in psoriasis by inhibiting the pyroptosis which NLRP3 mediated (16). In addition, previous research has established that aberrant NLRP3 inflammasome activation in masts cell contributes to histamine-independent urticaria by production of IL-1β in cryopyrin-associated periodic syndromes (CAPSs) (17). However, our understanding of CSU with pyroptosis is still pretty limited.

Microarray technology has been widely utilized into biological studies in recent years, and the data generated by it like the mRNA dataset could be an advantageous instrument for discovering critical factors of etiopathogenesis of diseases, which offers valuable insulation and foundation for further novel studies (18, 19). In this study, *via* applying the bioinformatic method, we analyzed the data of the Gene Expression Omnibus (GEO) (20), which has its origin in microarray technology, to explore immune cell infiltration and ceRNA network, and further reveal the molecular mechanism of pyroptosis-related genes in CSU and identify key biomarkers.

## Material and Method

### Microarray Data Source

**Figure 1:**
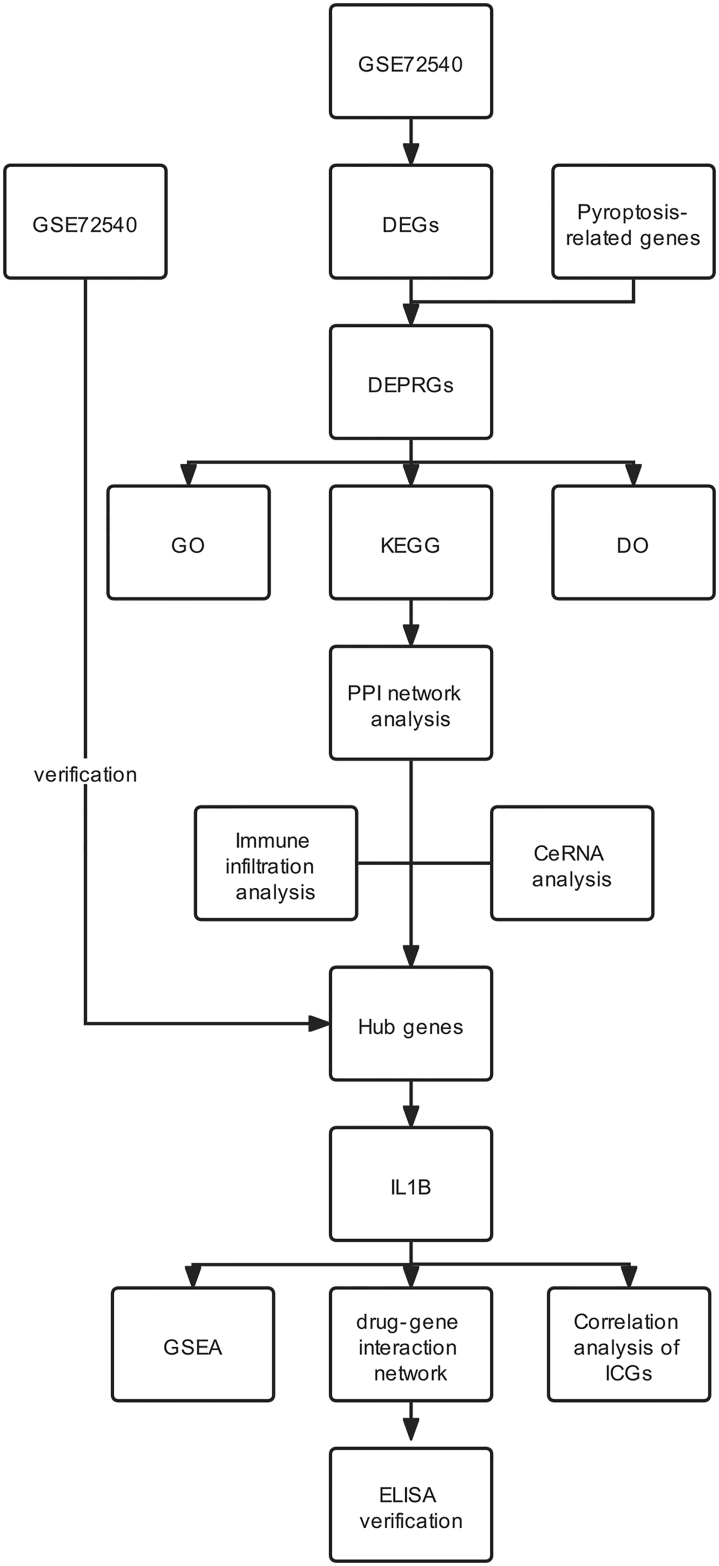
Flowchart of the study.

The analysis process of this study is shown in **Figure 1**. We downloaded the datasets (GSE72540, GSE57178) from the GEO database (**Table 1**). GSE72540 contained 31 samples’ RNA expression profiling, selecting 10 CSU samples and 8 control samples, and GSE57178 contained 18 samples, selecting 6 CSU samples and 7 control samples.

**Table 1:** Details of the GEO CSU data.

Dataset	Platform	Number of samples (CSU/control, subjects)
GSE72540	GPL16699	31 (10/8 18)
GSE57178	Agilent-039494 SurePrint G3 Human GE v2 8x60K Microarray 039381 (Feature Number version)	18 (6/7 13)
	GPL6244	
	[HuGene-1_0-st] Affymetrix Human Gene 1.0 ST Array [transcript (gene) version]	

GEO, Gene Expression Omnibus; CSU, chronic spontaneous urticaria.

### Identifying Differently Expressed Pyroptosis-Related Genes

We normalized and preprocessed data and identified the different expression genes (DEGs) among the CSU sample and control *via* the GEO2R tool (21). |log_2_ FC| >1 and p < 0.05 as the cutoff. The 161 pyroptosis-related genes (PRGs) were downloaded from the GeneCards database (**Supplementary Table S1**) (22). Altogether consistent genes between DEGs and PRGs were identified as differently expressed pyroptosis-related genes (DEPRGs).

### GO, KEGG, and DO Enrichment Analyses of DEPRGs

GO enrichment analysis [included MF (molecular function), BP (biological process), and CC (cellular component)] and KEGG pathway analysis were executed *via* the Metascape database (23). Min Enrichment ≥1.5, Min Overlap ≥3, and p < 0.01 were considered as the threshold. The WebGestalt tool (24) was used for DO enrichment analysis, and FDR ≤ 0.05 as the significance level.

### Protein–Protein Interaction Network and Module Analyses

To investigate the protein–protein interaction (PPI) network, we used the STRING tool (25) and visualized it and analyzed the interactions of DEPRGs by the Cytoscape software (26). The Molecular Complex Detection (MCODE) plug-in was utilized for the module analysis of the PPI network. The cytoHubba tool was used for identifying the hub genes. The hub genes’ GO enrichment analysis was performed through the ClueGO plug-in.

### Data Verification

The RNA expressed dataset GSE57178, containing 6 CSU lesion samples and 7 healthy control samples, was utilized as the validation set to verify the reliability of hub genes.

### Immune Infiltration Analysis

The immune infiltration was calculated by the web tool CIBERSORT (27), which is a deconvolution algorithm that can evaluate the proportion of 22 infiltrating lymphocyte subsets in a large number of tissue samples. The GraphPad Prism 8.0.2 (San Diego, CA, USA) tool (28) was utilized for the correlation analysis between different immune cells, and between immune cells and hub genes, calculating the ratio of every kind of immune cell in CSU tissue and control.

### Exploration ceRNA Network of the Hub Genes

To explore the miRNA–mRNA interaction of the ceRNA network, the potential miRNAs targeting the hub gene were identified *via* the TargetScan (29), miRNet (30), and DIANA TOOLS TarBase v.8 databases (31). If this was concurrently recognized in each database, the result was considered as true. Next, the possible lncRNAs targeting the miRNA were predicted through the miRNet database, which was cross-checked with the differently expressed lncRNA (DElncRNA) of CSU. LncRNA subcellular localization was predicted using lncLocator (32). The web-based tools, Wei Sheng Xin (http://www.bioinformatics.com.cn) and Draw Venn Diagram (http://bioinformatics.psb.ugent.be/webtools/Venn/), were used for data visualization.

### Gene Set Enrichment Analysis

The Gene Set Enrichment Analysis (GSEA) tool (33) was used for exploring the molecular signaling pathway in which IL1B might be involved in CSU. The pathway enrichment analysis utilized the c2.cp.kegg.v7.3.symbols.gmt gene sets of the official website. False discovery rate q-value <0.01 was regarded as difference.

### Analysis of Protein Subcellular Localization and Correlation With Immune Checkpoints

The protein subcellular localization of IL1B was predicted using the Cell-PLoc 2.0 tool (34), which is a package of web servers for predicting subcellular localization of proteins in different organisms. The correlation between IL1B and key immune checkpoints (35) such as HAVCR2(TIM3), LAG3, CTLA4, CD274(PDL1), PDCD1(PD1), and TIGIT were analyzed *via* Pearson’s correlation coefficient in GraphPad Prism 8.0.2.

### Drug–Gene Interaction and Molecular Docking Analysis

To explore the drug–gene interaction, the DrugBank database (36) was utilized for identifying existing or/and potentially associated drug substances. Moreover, the Cytoscape software was utilized for data visualization. The molecular structure of the ligand and the target protein were obtained from the PubChem database (37) and PDB database (38). Docking simulations were conducted through AutoDock Vina (39) to generate the docking energy. The PyMOL software (40) was performed to visualize docked complexes.

### Enzyme-Linked Immunosorbent Assay

To examine the protein levels of IL1B, NLRP3, and mast cell tryptase (MCT), serums from 10 CSU patients and 10 healthy controls were harvested for enzyme-linked immunosorbent assay (ELISA) (the CSU patients without medical treatment within 2 weeks and concomitant autoimmune diseases, whose detailed information is in **Supplementary Table S2**). Specific ELISA kits for IL1B (Neobioscience, Shenzhen, China), NLRP3 (uscnk, Wuhan, China), and MCT (Fufeng, Shanghai, China) were used according to the instructions of the manufacturer. Briefly, the standard samples, which were offered through the kit, of known concentration and the samples from two experimental groups were added to the kit plate and then incubated with the kit reagents (41). The OD450 values were detected using the microplate reader (Huisong, Shenzhen, China).

### Statistical Analysis

The unpaired Student’s t-test was performed for data analysis of two groups. The potential correlation between the two variables was detected by Pearson’s correlation coefficient. p < 0.05 was considered as significance level. GraphPad Prism 8.0.2 was performed as the statistical software.

## Results

### Recognition of DEPRGs of CSU

**Figure 2:**
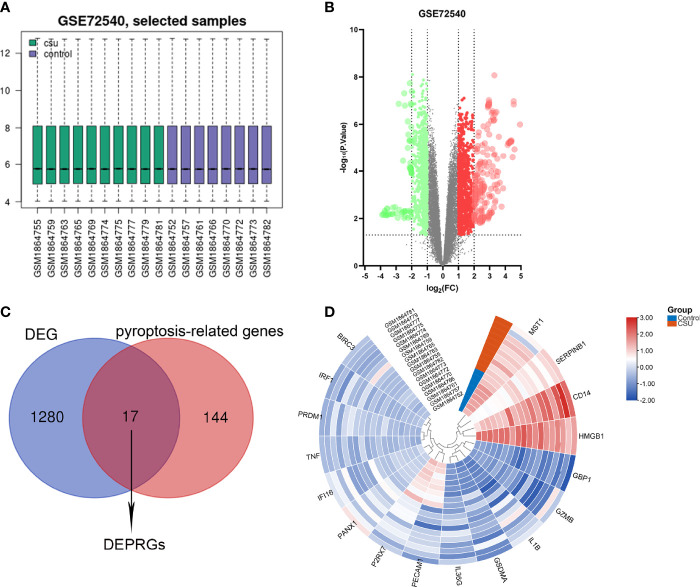
Identification of DEPRGs of CSU. **(A)** Normalization of selected samples of GSE72540. **(B)** The differentially expressed genes of GSE72540. **(C)** The DEPRGs of CSU. **(D)** The heat map of the DEPRGs.

The CSU RNA expression profile dataset (GSE72540) was normalized as shown in **Figure 2A**. 1,297 DEGs (containing 1,033 DEmRNAs and 173 DElncRNAs) were identified in the GSE72540 dataset (**Supplementary Table S3**), and their volcano plot is shown in **Figure 2B**. As shown in **Figure 2C** and **Table 2**, we identified 17 congruous DEPRGs *via* integrated bioinformatics analysis, including 13 congruously upregulated and 4 congruously downregulated. The heat map of DEPRGs is shown in **Figure 2D**.

**Table 2:** The DEPRGs of CSU.

Regulation	DEPRGs
Upregulated (n = 13)	SERPINB1 GBP1 IFI16 GSDMA GZMB BIRC3 IL1B IRF1 CD14 PRDM1 IL36G PANX1 TNF
Downregulated (n = 4)	PECAM1 P2RX7 MST1 HMGB1

DEPRGs, differently expressed pyroptosis-related genes; CSU, chronic spontaneous urticaria.

### Function Enrichment Analyses of the DEPRGs

**Figure 3:**
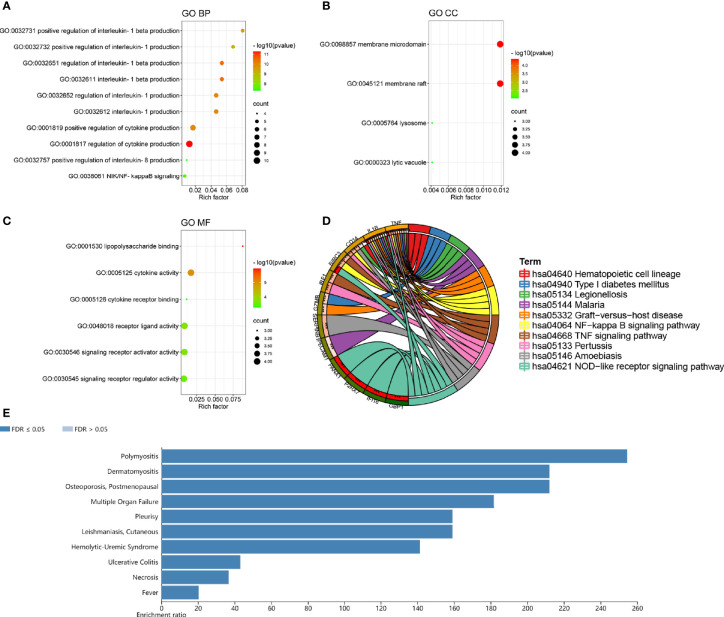
The top ten lists of function enrichment analyses of DEPRGs. **(A)** GO BP; **(B)** GO CC; **(C)** GO MF; **(D)** KEGG signaling pathway; **(E)** DO enrichment. **p < 0.01; ***p < 0.001.

The GO analysis of DEPRGs was performed to reveal their biology functions. As shown, in the GO BP category, most of DEPRGs were mostly involved into regulation of cytokine production, interleukin-1 beta production, interleukin-1 production, etc. (**Figure 3A**). In the GO CC category, most of the DEPRGs were enriched into the membrane microdomain and membrane raft (**Figure 3B**). In the GO MF category, the main DEPRGs were enriched in cytokine activities, cytokine receptor binding, and signaling receptor activator activity, etc. (**Figure 3C**). The results of KEGG pathway enrichment exhibited that the mostly involved pathways were the NF-kappa B signaling pathway, TNF signaling pathway, and NOD-like receptor signaling pathway (**Figure 3D**). Utilizing the WebGestalt online database to further explore the function of DEPRGs, the result of DO enrichment showed that dermatomyositis, leishmaniasis, cutaneous, ulcerative colitis, etc., were the major diseases that DEPRGs participated in (**Figure 3E**). These suggested that inflammation and immune response were the major function of DEPRGs.

### PPI Network and Hub Gene Analyses

**Figure 4:**
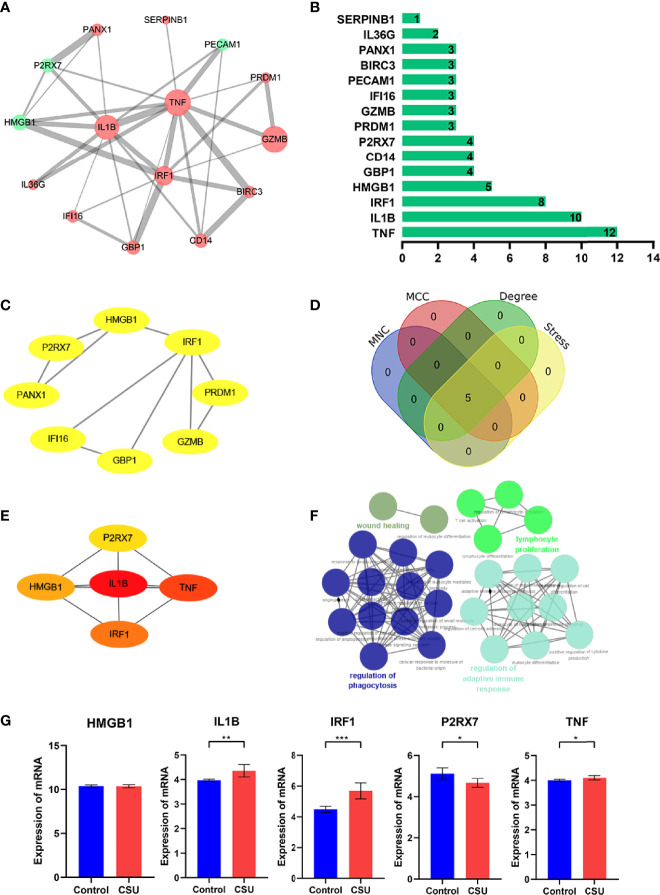
The PPI network and hub gene analyses. **(A)** The PPI network of the DEPRGs, the bigger sizes of the edge and node mean the higher degree. The red means upregulated, and green means downregulated. **(B)** The connectivity rank of genes. **(C)** The first module of the PPI network. **(D)** Four algorithms were utilized to identified hub genes. **(E)** 5 hub genes of CSU. **(F)** The biological process of hub genes *via* the ClueGO tool. **(G)** Data validation of hub genes by GSE57178. *P < 0.05; **P < 0.01; ***P < 0.001.

To reveal the interaction of each protein, the PPI network of the DEPRGs was built according to the STRING database, including 15 nodes and 34 edges. In the protein network graph, each node represented a protein, and the edge represented a connection between two proteins. Moreover, among the 15 nodes, 3 nodes were downregulated, and 12 were upregulated (**Figure 4A**). The targets were sorted by target connectivity from small to large in the PPI network, as shown in **Figure 4B**. The most important module was selected, including 10 edges and 8 nodes (**Figure 4C**). Hub genes were detected consistently *via* four algorithms (degree, MNC, stress, and MCC) of cytoHubba (**Figure 4D**). The top five gene scores were considered to be hub genes of CSU: IL1B, TNF, IRF1, HMGB1, and P2RX7 (**Figure 4E** and **Table 3**). Because the more closely knitted gene in the network is more fundamental to regulation, we further investigated the functions of the hub genes through the ClueGO plug-in. As **Figure 4F** shows, they were still primarily enriched in immunomodulation including regulation of adaptive immune response, lymphocyte proliferation, and regulation of phagocytosis. According to GSE57178, the mRNA expression of each hub gene manifested that, compared with the control, IL1B, IRF1, and TNF were significantly overexpressed while P2RX7 had a significantly lower expression in CSU, which was the same as the above results and indicated that IL1B, IRF1, P2RX7, and TNF were the key genes of CSU (**Figure 4G**).

**Table 3:** The top 5 hub genes.

Genes	Description	Degree	MCC	MNC	Sterss	Log_2_FC	Expression change
IL1B	Interleukin 1 beta	10	38	10	78	3.43222906	Upregulated
TNF	Tumor necrosis factor	12	37	11	130	1.26641463	Upregulated
IRF1	Interferon regulatory factor 1	8	26	8	48	1.17112479	Upregulated
HMGB1	High mobility group box 1	5	18	5	20	-1.01820995	Downregulated
P2RX7	Purinergic receptor P2X 7	4	12	4	10	-1.25950113	Downregulated

MCC, maximal clique centrality; MNC, maximum neighborhood component.

### Immune Infiltration Analysis

**Figure 5:**
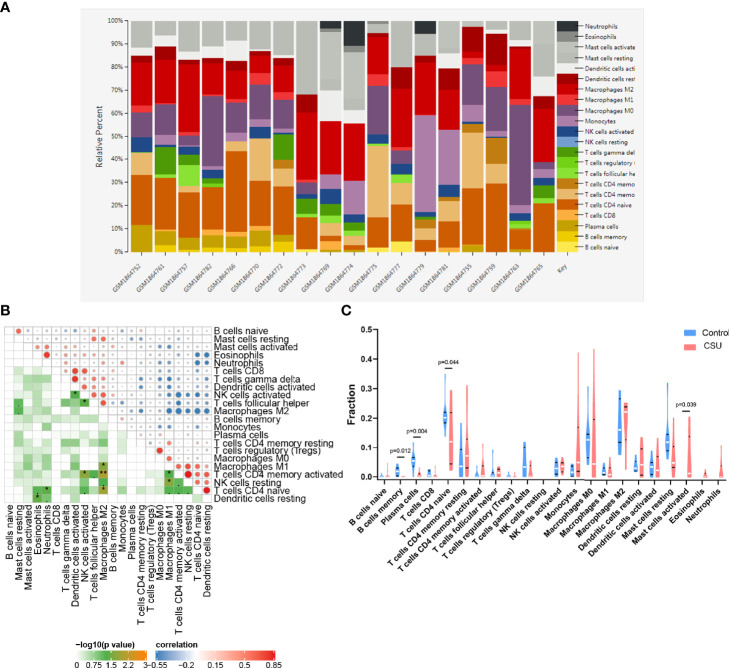
Immune infiltration analysis of CSU. **(A)** The ratio of 22 immune cells of each sample of CSU. **(B)** The correlation between each of immune cells. **(C)** The proportion of immune cells in CSU and control.

**Figure 6:**
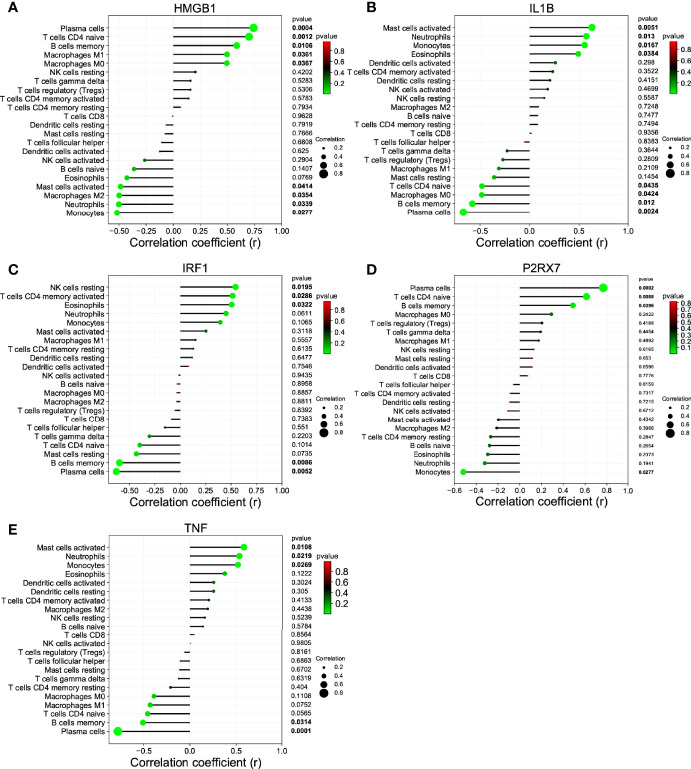
The correlation between the hub gene and the immune cell. **(A)** HMGB1; **(B)** IL1B; **(C)** IRF1; **(D)** P2RX7; and **(E)** TNF.

We investigated the difference among CSU tissues and control to explore the panorama of immune infiltration of CSU *via* the CIBERSORT algorithm. The ratio of 22 immune cells of samples is shown in **Figure 5A**. The correlation between each of immune cells is shown in **Figure 5B**, among which T cells CD4 memory activated were significantly correlated with macrophages M1 and macrophages M2, and eosinophils were statistically correlated with dendritic cells resting. At the side of control tissue, CSU owned a higher ratio of mast cells activated, and T cells CD4 naive, plasma cells, and B cells memory were significantly lower (**Figure 5C**). Next, we revealed the relation among the abundance of the immune cells and hub gene expression through the Pearson’s correlation coefficient. The results displayed that mast cells activated were statistically positively related to the levels of IL1B and TNF, but negatively to HMGB1’s; B cells memory and plasma cells were positively correlated with HMGB1 and P2RX7, but negatively with IL1B, IRF1, and TNF; T cells CD4 naive were positively correlated with HMGB1 and P2RX7, but negatively with IL1B (**Figure 6**).

### The mRNA–miRNA–lncRNA ceRNA Network of CSU

**Figure 7:**
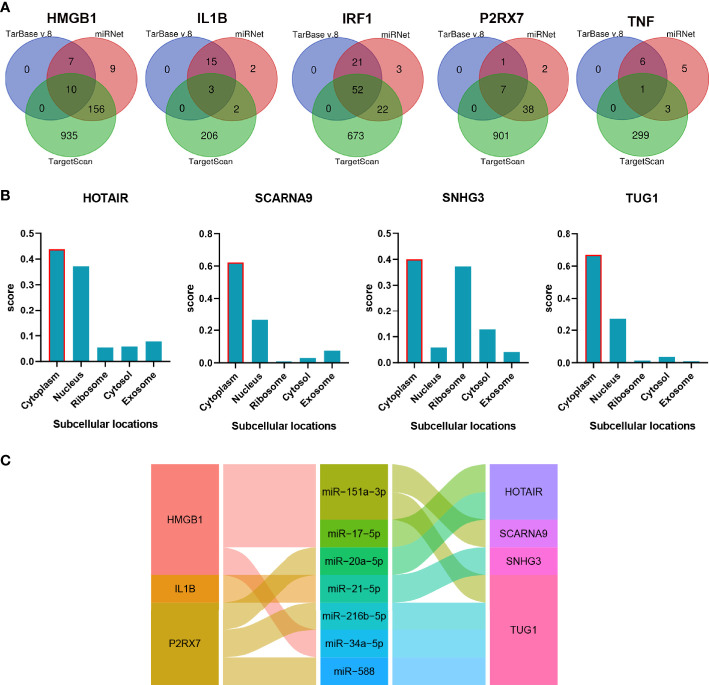
The construction of the lncRNA–miRNA–mRNA ceRNA network of CSU. **(A)** Venn diagram of miRNAs targeting each hub gene. **(B)** The subcellular localization of lncRNA of ceRNA. **(C)** The alluvial diagram of the ceRNA network.

**Figure 8:**
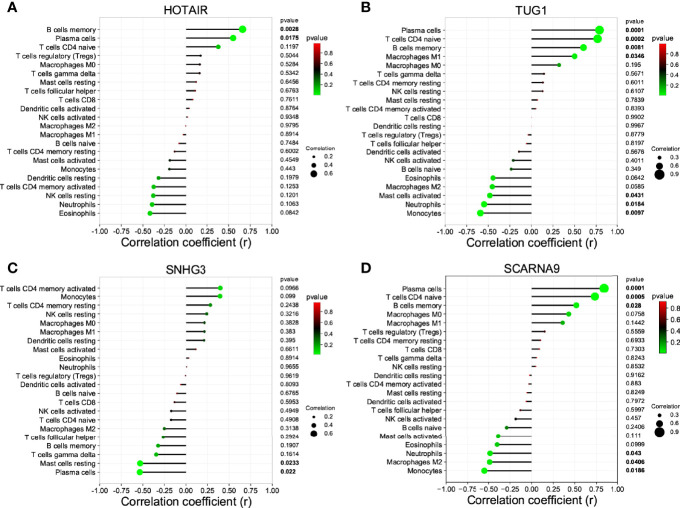
The correlation between the lncRNA of ceRNA and immune cells. **(A)** HOTAIR; **(B)** TUG1; **(C)** SNG3; **(D)** SCARNA9.

The non-coding RNA (ncRNA) never participates in encoding proteins but was discovered to be involved in many biological functions, and perturbation of mRNA–miRNA–lncRNA ceRNA networks may affect diseases. The miRNA-targeting hub gene was concurrently recognized by all object databases as true, and their Venn diagrams are shown in **Figure 7A**. In addition, the possible lncRNA targeting the miRNA was predicted *via* miRNet online databases and got the intersection with 173 DElncRNAs. The lncRNA and mRNA of ceRNA must have a consistent expression trend according to the ceRNA mechanism. Then, we got 9 unique DElncRNAs based on the above. The lncRNAs, which compete with miRNAs by acting as ceRNAs to regulate the expression of mRNA targets, should be in the cytoplasm. As a result, only 4 DElncRNAs (HOTAIR, SCARNA9, SNHG3, and TUG1) were predicted in the cytoplasm by lncLocator (**Figure 7B**). Finally, an 18-axis ceRNA network (containing HMGB1/hsa-mir-17-5p/HOTAIR, IL1B/hsa-mir-21-5p/SNHG3, P2RX7/hsa-mir-20a-5p/HOTAIR and so on) was identified (**Figure 7C**). In addition, HOTAIR, SCARNA9, SNHG3, and TUG1 were significantly related to the major infiltration cell of CSU (**Figure 8**).

### GSEA of IL1B

**Figure 9:**
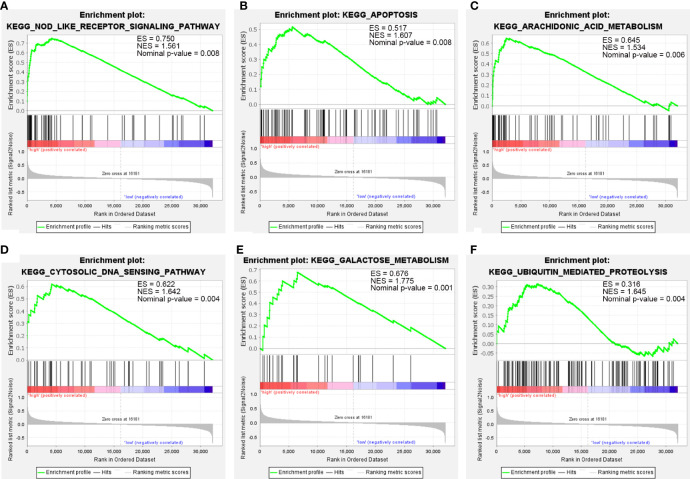
The GSEA of IL1B. **(A)** NOD-like receptor signaling pathway. **(B)** Apoptosis. **(C)** Arachidonic acid metabolism. **(D)** Cytosolic DNA sensing pathway. **(E)** Galactose metabolism. **(F)** Ubiquitin-mediated proteolysis.

Due to the fact that IL1B had been verified and that it played a role in immune infiltration and the ceRNA network of CSU, and simultaneously log_2_FC of IL1B was maximal in the hub genes, we chose IL1B for further analysis. The result of GSEA further verified the above results. As **Figure 9** shows, besides ubiquitin-mediated proteolysis, arachidonic acid metabolism, cytosolic DNA sensing pathway, galactose metabolism, and apoptosis, IL1B was still mainly enriched in the NOD-like receptor signaling pathway.

### Protein Subcellular Localization and Correlation With Immune Checkpoint Analyses of IL1B

**Figure 10:**
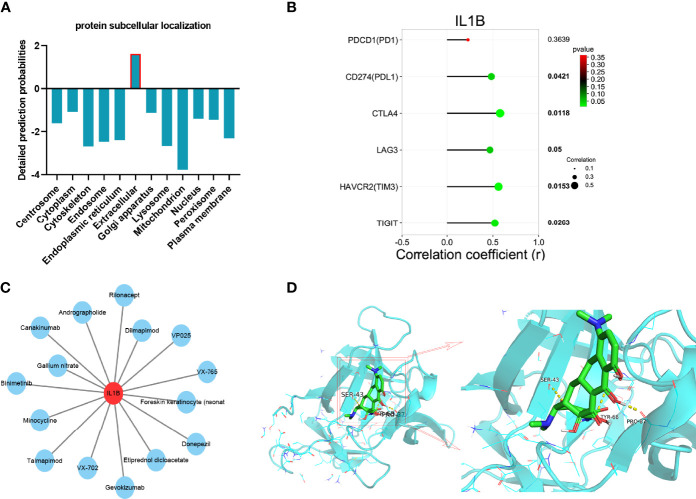
Integrated analyses of IL1B. **(A)** protein subcellular localization of IL1B. **(B)** The correlation between immune checkpoints and IL1B. **(C)** Drug–gene interaction network of IL1B. **(D)** Molecular docking between IL1B and minocycline.

Different subcellular localizations of protein decide different biological functions. The protein subcellular localization of IL1B predicted by Cell-PLoc 2.0 was extracellular (**Figure 10A**). As displayed in **Figure 10B**, IL1B was significantly correlated with familiar immune checkpoints such as CD274(PDL1), CTLA4, HAVCR2(TIM3), and TIGIT, which indicated the important effect of IL1B in immune response further.

### Drug–Gene Interaction and Molecular Docking Analyses of IL1B

Developing potential therapeutic drugs for targeting IL1B provides a specific treatment strategy. The drug–gene interaction network of IL1B is exhibited in **Figure 10C**, in which there were 15 potential therapeutic drugs identified and 7 of them were approved (**Table 4**). Then, we worked out the molecular binding site of IL1B and minocycline, one of approved small-molecule drugs (**Figure 10D**).

**Table 4:** The drugs approved to interact IL1B.

DrugBank ID	Name	Pharmacological action	Actions
DB01017	Minocycline	Unknown	Modulator
DB00843	Donepezil	Unknown	Inhibitor inducer
DB10772	Foreskin keratinocyte (neonatal)	Yes	Agonist
DB06168	Canakinumab	Yes	Binder
DB06372	Rilonacept	Unknown	Binder
DB05260	Gallium nitrate	Yes	Antagonist
DB11967	Binimetinib	Unknown	/

### IL1B Might Participate in Activation of Mast Cells *via* the NLRP3 in CSU

**Figure 11:**
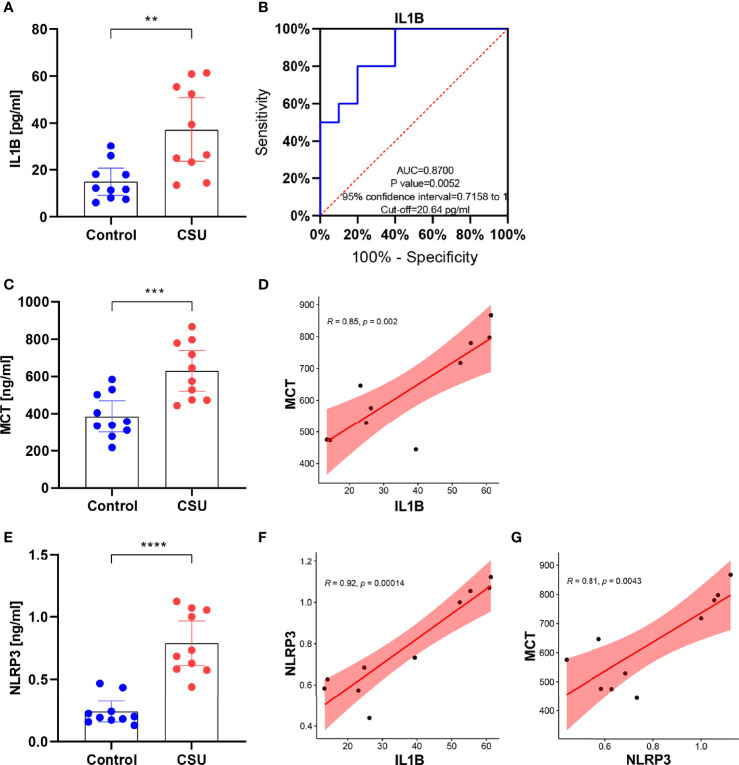
IL1B might participate in activation of mast cells *via* the NLRP3 in CSU. **(A)** The expression of IL1B in CSU and control. **(B)** The ROC curve of IL1B. **(C)** The expression of MCT in CSU and control. **(D)** The correlation between IL1B and MCT in CSU. **(E)** The expression of NLRP3 in CSU and control. **(F)** The correlation between IL1B and NLRP3 in CSU. **(G)** The correlation between NLRP3 and MCT in CSU. **p < 0.01, ***p < 0.001, ****p < 0.0001.

Compared with the healthy control, IL1B showed a significant overexpression in serum of CSU patients *via* ELISA, which is the same as our bioinformatic prediction (**Figure 11A**). The result of the ROC curve analysis showed that the area under the curve was 0.87 (p 0.01), which suggested the role of IL1B in diagnosis of CSU and further that it may be a potential biomarker in CSU (**Figure 11B**). Moreover, the result exhibited that MCT was overexpressed in CSU, and expression of MCT was statistically correlated with IL1B (**Figures 11C, D** **)**. Due to the fact that MCT is the key marker of mast cell activation (42), it indicated that IL1B may participate in mast cell activation. NLRP3 is a subtype of NOD-like receptors and is famous for one of the key pyroptosis cytokines (43). The further ELISA result of CSU patient serum displayed that NLRP3 was significantly overexpressed and correlated with IL1B and MCT (**Figures 11E–G**). It testified the above bioinformatic prediction again which IL1B participated in, in the NOD-like receptor signaling pathway. Moreover, it also advised that it could be *via* the NLRP3 that IL1B participates in activation of mast cells.

## Discussion

CSU is a common chronic inflammatory dermatosis, which has significantly negative impacts on the quality of people’s life owing to its repeated outbreaks and protracted course (44). Although current treatments of CSU get little effectiveness, how to more effectively mitigate and avert recurrence is still a global challenge as there are still many unknowns in its genesis. In addition, it has reached a consensus in the last guidelines that further research in some areas of CSU is needed, such as identification of mast cell/basophil-activating factors, identification of serum biomarkers of urticarial activity/mast cell activation, and identification of new histological markers (1). Remarkably, pyroptosis was one of deaths associated with cell membrane rupture. The increased number of cell membranes in mast cells might lead to the liberation of intracellular β-hexosaminidase and histamine (45). Since pyroptosis showed a great research prospect in inflammatory skin diseases, this work tries to identify and verify the potential key biomarkers of CSU from the standpoint of pyroptosis-related genes through bioinformatics ways, especially in inflammatory response, to provide a new perspective for the etiopathogenesis and therapeutic approaches of CSU.

In the present research, we recognized 1,297 DEGs from the CSU RNA expression profile. Then crossing the DEGS with pyroptosis-related genes, 17 DEPRGs (containing 4 downregulated genes and 13 upregulated genes) were recognized and then performed into gene function analysis. As shown, the DEPRGs were mostly involved in inflammatory response, as well as in pro-inflammatory effects (such as positive regulation of interleukin-8 production, positive regulation of interleukin-1 beta production, positive regulation of interleukin-1 production) and biological regulation (including signaling receptor activator activity, receptor ligand activity, cytokine activity), the majority of which are generally accredited to constituent parts of the development of CSU. A research containing 153 CSU patients suggested that the IL1 gene had a significant role in the susceptibility to CSU (46). Kasperska-Zajac et al. indicated that severity of systemic inflammation of CSU was related to elevated il-8 (47). The DEPRGs mainly participated in inflammatory pathways according to KEGG, likely the NF-kappa B signaling pathway, TNF signaling pathway, and NOD-like receptor signaling pathway. In addition, the result of DO further confirms the above. The DEPRGs were majorly enriched in inflammatory diseases like ulcerative colitis and fever. This advises that, to some extent, the DEPRGs could have a function to participate in the systemic inflammation of CSU.

Through the PPI network and module analyses, we identified five hub genes, namely, IL1B, TNF, IRF1 (all upregulated genes) and HMGB1, P2RX72 (both downregulated genes). They are the common inflammatory cytokines, but most of them have not been reported to be implicated in the development of CSU, so this would be a new finding. To fully explore the maladjustment of inflammatory cells of CSU, we executed immune infiltration analysis. It was found that CSU tissue owned a higher proportion of mast cells activated, but relatively lower ones of T cells CD4 naive, plasma cells, and B cells memory. Previous studies indicated that CSU was considered to be principally a mast cell-driven disease (48). Moreover, it has also been reported that the etiopathogenesis of CSU was closely associated with the dynamical unbalance of Th1/Th2 cells of CD4+T cells (49). However, since there have been few research reports, the relationship between CSU and plasma cells or/and B cells memory might be an interesting finding. Moreover, our research further showed that each of the hub genes (IL1B, TNF, IRF1, HMGB1, and P2RX7) was statistically related to major infiltration cells. Especially, IL1B and TNF were statistically and positively associated with mast cells activated, which suggests that they are related to maladjustment of inflammatory cells of CSU and might be its possible immunomodulation pivots. In addition, to reveal a systemically interactive modulation in CSU, we constructed a ceRNA network, in which there were 8 axes, such as IL1B/miR-21-5p/SNHG3, HMGB1/miR-34a-5p/TUG1, and P2RX7/miR-588/TUG1. It is worth noting that we also found that SNHG3 was significantly and negatively correlated with mast cells resting and plasma cells, and TUG1 was negatively related to mast cells activated, which further verified that the DEmRNA–miRNA–DElncRNA ceRNA network did have a critical role in maladjustment of inflammatory cells of CSU.

We chose IL1B to do further analysis for three reasons. First, it had the biggest fold change in hub genes. Second, it was verified by the validation set GSE57178. Third, both it and its upstream factor SNHG3 were related to the activation of mast cells. These indicated that IL1B could be in a more critical position in the development of CSU. IL1B (IL-1β) is a potent pro-inflammatory cytokine and plays a role in the innate and adaptive immunity of humans (50). Under the stimulation of immune response, inflammation, and infection, IL1B is released from monocytes, macrophages, and dendritic cells, affects local cells by paracrine, and targets distant cells *via* endocrine, ultimately leading to a series of inflammatory cascade responses like activation of immune cells and pyroptosis (51). Abnormal IL1B-related signaling pathways have been shown to be connected with some immune inflammatory diseases like SLE and UC (52, 53). These were in agreement with the result of our research. We predicted that the protein subcellular localization of IL1B was extracellular, which is in accord with its intercellular regulation role. Moreover, it was found that IL1B was significantly correlated with familiar immune checkpoints in CSU, such as PDL1, CTLA4, TIM3, and TIGIT, which someway showed its role of immune regulation in CSU. Furthermore, IL1B was an overexpression examined in clinical CSU patients by ELISA, and the ROC curve analysis confirmed the dependability of its diagnostic value; the point was that mast cells were significantly activated in CSU and IL1B did correlate with it, which verified our bioinformatic analyses and suggests that IL1B could be a potential prognostic and diagnostic biomarker in CSU.

The NOD-like receptor signaling pathway was the major involved pathway according to the enrichment analysis of IL1B in a couple of databases. Moreover, NLRP3 is a subtype of NOD-like receptors and is famous for one of key pyroptosis cytokines; moreover, it is a well-known activator of IL1B (54). The study of Guo et al. showed that the increased expression of NLRP3 in mast cells leads to the activation of caspase-1 and ultimately to production and secretion of IL-1β in endometriosis (55). These are further supported in our work. In clinical CSU patients, NLRP3 was statistically overexpressed and related to the activation of mast cells. NLRP3 was also significantly correlated with IL1B, which might advise that the pyroptosis-related signaling pathway is activated in CSU and it might be related to the activation of mast cells. What is more, it might be *via* the NOD-like receptor signaling pathway, NLRP3, that IL1B participates in activation of mast cells. Moreover, we further identified 15 potential therapeutic drugs targeting IL1B, which provides a possible therapeutic strategy for CSU. Molecular docking revealed that the exact molecular binding makes this relationship more reliable.

Our study also had some limitations. We measured gene expression levels using sera from clinical CSU patients rather than tissues, which is not good enough but still clinically representative. Besides, we will perform the experiments *in vivo* and *in vitro* to further confirm our results in the future.

In sum, we identified 5 hub genes, IL1B, TNF, IRF1, HMGB1, and P2RX7, from pyroptosis-related genes, which are mainly involved in the inflammatory response and maladjustment of inflammatory cells of CSU. Particularly, ILB and its ceRNA axis might play a role in the activation of mast cells of CUS, and this might be achieved *via* the NOD-like receptor signaling pathway (NLRP3). Therefore, ILB and its related molecules might be potential key biomarkers in the development of CSU, and our study would provide a new perspective for the etiopathogenesis and therapeutic programs of CSU.

## Data Availability Statement

The datasets presented in this study can be found in online repositories. The names of the repository/repositories and accession number(s) can be found in the article/**Supplementary Material**.

## Ethics Statement

Written informed consent was obtained from the individual(s)’ and minor(s)’ legal guardian/next of kin, for the publication of any potentially identifiable images or data included in this article.

## Author Contributions

SP and HF conceptualized the study design. SP and TZ drafted the manuscript. HF revised the manuscript. SP, SZ, QT, and YY collected data and performed the analysis. All authors contributed to the article and approved the submitted version.

## Funding

This work was financed by the Hunan Provincial Health Commission scientific research projects (B2016229; B2019055; 202114051780) and Hunan Provincial Innovation Foundation for Postgraduate (CX20200547).

## Conflict of Interest

The authors declare that the research was conducted in the absence of any commercial or financial relationships that could be construed as a potential conflict of interest.

## Publisher’s Note

All claims expressed in this article are solely those of the authors and do not necessarily represent those of their affiliated organizations, or those of the publisher, the editors and the reviewers. Any product that may be evaluated in this article, or claim that may be made by its manufacturer, is not guaranteed or endorsed by the publisher.

## Supplementary Material

Supplementary Table S1 161 pyroptosis-related genes

Supplementary Table S2 Population characteristics

Supplementary Table S3 1033 DEmRNAs and 173 DElncRNAs of CSU

Supplementary Table S4 The DEGs of GSE72540

Supplementary Table S5 The GO and KEGG enrichment of DEPRGs

Supplementary Table S6 PPI topology table

Supplementary Table S7 Hub genes-mirRNAs

Supplementary Table S8 MiRNAs-lncRNAs
