# Supplementary material for: Integrated Bioinformatics and Validation Reveal IL1B and Its Related Molecules as Potential Biomarkers in Chronic Spontaneous Urticaria
Source: Front Immunol. 2022 Mar 18;13:850993. doi: 10.3389/fimmu.2022.850993 (PMC8975268; doi:10.3389/fimmu.2022.850993)
Supplement: Supplementary Table S1 — 161 pyroptosis-related genes [file Table_1.docx]

| Gene Symbol | Description |
| --- | --- |
| GSDMD | Gasdermin D |
| GSDME | Gasdermin E |
| NLRP3 | NLR Family Pyrin Domain Containing 3 |
| CASP1 | Caspase 1 |
| GSDMB | Gasdermin B |
| CASP4 | Caspase 4 |
| GSDMC | Gasdermin C |
| NLRP1 | NLR Family Pyrin Domain Containing 1 |
| GSDMA | Gasdermin A |
| CARD8 | Caspase Recruitment Domain Family Member 8 |
| GZMB | Granzyme B |
| IL1B | Interleukin 1 Beta |
| GZMA | Granzyme A |
| NLRC4 | NLR Family CARD Domain Containing 4 |
| CASP8 | Caspase 8 |
| CASP5 | Caspase 5 |
| AIM2 | Absent In Melanoma 2 |
| ZBP1 | Z-DNA Binding Protein 1 |
| PYCARD | PYD And CARD Domain Containing |
| NAIP | NLR Family Apoptosis Inhibitory Protein |
| DHX9 | DExH-Box Helicase 9 |
| NLRP9 | NLR Family Pyrin Domain Containing 9 |
| CASP3 | Caspase 3 |
| IL18 | Interleukin 18 |
| HMGB1 | High Mobility Group Box 1 |
| APIP | APAF1 Interacting Protein |
| KCNQ1OT1 | KCNQ1 Opposite Strand/Antisense Transcript 1 |
| MIR223 | MicroRNA 223 |
| TREM2 | Triggering Receptor Expressed On Myeloid Cells 2 |
| FOXO3 | Forkhead Box O3 |
| MALAT1 | Metastasis Associated Lung Adenocarcinoma Transcript 1 |
| CASP6 | Caspase 6 |
| TXNIP | Thioredoxin Interacting Protein |
| MIR22 | MicroRNA 22 |
| DDX3X | DEAD-Box Helicase 3 X-Linked |
| MIR125A | MicroRNA 125a |
| GBP1 | Guanylate Binding Protein 1 |
| GJA1 | Gap Junction Protein Alpha 1 |
| MIR30C1 | MicroRNA 30c-1 |
| MIR214 | MicroRNA 214 |
| PRDM1 | PR/SET Domain 1 |
| CPTP | Ceramide-1-Phosphate Transfer Protein |
| CRTAC1 | Cartilage Acidic Protein 1 |
| NEK7 | NIMA Related Kinase 7 |
| TP53 | Tumor Protein P53 |
| AGER | Advanced Glycosylation End-Product Specific Receptor |
| CTSV | Cathepsin V |
| MIR155 | MicroRNA 155 |
| NFE2L2 | Nuclear Factor, Erythroid 2 Like 2 |
| TET2 | Tet Methylcytosine Dioxygenase 2 |
| APOE | Apolipoprotein E |
| CD274 | CD274 Molecule |
| EEF2K | Eukaryotic Elongation Factor 2 Kinase |
| FGF21 | Fibroblast Growth Factor 21 |
| KLF3-AS1 | KLF3 Antisense RNA 1 |
| NFKB1 | Nuclear Factor Kappa B Subunit 1 |
| P2RX7 | Purinergic Receptor P2X 7 |
| SDHB | Succinate Dehydrogenase Complex Iron Sulfur Subunit B |
| CEBPB | CCAAT Enhancer Binding Protein Beta |
| IL32 | Interleukin 32 |
| MEG3 | Maternally Expressed 3 |
| MIR135B | MicroRNA 135b |
| MIR21 | MicroRNA 21 |
| MIR485 | MicroRNA 485 |
| TFAM | Transcription Factor A, Mitochondrial |
| MALT1 | MALT1 Paracaspase |
| CDKN2B-AS1 | CDKN2B Antisense RNA 1 |
| ELAVL1 | ELAV Like RNA Binding Protein 1 |
| MIR497 | MicroRNA 497 |
| MIR9-1 | MicroRNA 9-1 |
| MIR9-2 | MicroRNA 9-2 |
| MIR9-3 | MicroRNA 9-3 |
| MST1 | Macrophage Stimulating 1 |
| PRF1 | Perforin 1 |
| STK4 | Serine/Threonine Kinase 4 |
| HDAC6 | Histone Deacetylase 6 |
| HNP1 | Hypertensive Nephropathy |
| IRF3 | Interferon Regulatory Factor 3 |
| SQSTM1 | Sequestosome 1 |
| STING1 | Stimulator Of Interferon Response CGAMP Interactor 1 |
| METTL3 | Methyltransferase 3, N6-Adenosine-Methyltransferase Complex Catalytic Subunit |
| MIR25 | MicroRNA 25 |
| PECAM1 | Platelet And Endothelial Cell Adhesion Molecule 1 |
| PTEN | Phosphatase And Tensin Homolog |
| CAMP | Cathelicidin Antimicrobial Peptide |
| MRE11 | MRE11 Homolog, Double Strand Break Repair Nuclease |
| GBP5 | Guanylate Binding Protein 5 |
| PARP1 | Poly(ADP-Ribose) Polymerase 1 |
| NR1H2 | Nuclear Receptor Subfamily 1 Group H Member 2 |
| CTSG | Cathepsin G |
| MKI67 | Marker Of Proliferation Ki-67 |
| IL36B | Interleukin 36 Beta |
| IL36G | Interleukin 36 Gamma |
| PRTN3 | Proteinase 3 |
| SERPINB1 | Serpin Family B Member 1 |
| APOL1 | Apolipoprotein L1 |
| ANO6 | Anoctamin 6 |
| BNIP3 | BCL2 Interacting Protein 3 |
| MIR103A1 | MicroRNA 103a-1 |
| MIR103A2 | MicroRNA 103a-2 |
| NLRP7 | NLR Family Pyrin Domain Containing 7 |
| XIST | X Inactive Specific Transcript |
| FADD | Fas Associated Via Death Domain |
| SESN2 | Sestrin 2 |
| CAPN1 | Calpain 1 |
| JUN | Jun Proto-Oncogene, AP-1 Transcription Factor Subunit |
| MIR139 | MicroRNA 139 |
| TNF | Tumor Necrosis Factor |
| VIM | Vimentin |
| MEFV | MEFV Innate Immuity Regulator, Pyrin |
| ADAMTS9-AS2 | ADAMTS9 Antisense RNA 2 |
| ALK | ALK Receptor Tyrosine Kinase |
| BIRC2 | Baculoviral IAP Repeat Containing 2 |
| BIRC3 | Baculoviral IAP Repeat Containing 3 |
| GLMN | Glomulin, FKBP Associated Protein |
| IRGM | Immunity Related GTPase M |
| LY96 | Lymphocyte Antigen 96 |
| NLRP13 | NLR Family Pyrin Domain Containing 13 |
| RIPK3 | Receptor Interacting Serine/Threonine Kinase 3 |
| SIRT1 | Sirtuin 1 |
| UBE2D2 | Ubiquitin Conjugating Enzyme E2 D2 |
| TUBB6 | Tubulin Beta 6 Class V |
| NOS1 | Nitric Oxide Synthase 1 |
| NOS2 | Nitric Oxide Synthase 2 |
| PYDC2 | Pyrin Domain Containing 2 |
| ACE2 | Angiotensin Converting Enzyme 2 |
| AKT1 | AKT Serine/Threonine Kinase 1 |
| ATF6 | Activating Transcription Factor 6 |
| CASP9 | Caspase 9 |
| EGFR | Epidermal Growth Factor Receptor |
| IFI16 | Interferon Gamma Inducible Protein 16 |
| IRF1 | Interferon Regulatory Factor 1 |
| IRF2 | Interferon Regulatory Factor 2 |
| ORMDL3 | ORMDL Sphingolipid Biosynthesis Regulator 3 |
| POP1 | POP1 Homolog, Ribonuclease P/MRP Subunit |
| TP63 | Tumor Protein P63 |
| ANXA2 | Annexin A2 |
| BCL2 | BCL2 Apoptosis Regulator |
| BECN1 | Beclin 1 |
| BST2 | Bone Marrow Stromal Cell Antigen 2 |
| BTK | Bruton Tyrosine Kinase |
| CD14 | CD14 Molecule |
| CHI3L1 | Chitinase 3 Like 1 |
| CLEC5A | C-Type Lectin Domain Containing 5A |
| CXCL8 | C-X-C Motif Chemokine Ligand 8 |
| GAS5 | Growth Arrest Specific 5 |
| GPER1 | G Protein-Coupled Estrogen Receptor 1 |
| GSTO1 | Glutathione S-Transferase Omega 1 |
| HUWE1 | HECT, UBA And WWE Domain Containing E3 Ubiquitin Protein Ligase 1 |
| IL13 | Interleukin 13 |
| IL13RA2 | Interleukin 13 Receptor Subunit Alpha 2 |
| IL1RN | Interleukin 1 Receptor Antagonist |
| LRPPRC | Leucine Rich Pentatricopeptide Repeat Containing |
| LYST | Lysosomal Trafficking Regulator |
| MDM2 | MDM2 Proto-Oncogene |
| MIR15A | MicroRNA 15a |
| MIR20B | MicroRNA 20b |
| NCR1 | Natural Cytotoxicity Triggering Receptor 1 |
| PANX1 | Pannexin 1 |
| STAT3 | Signal Transducer And Activator Of Transcription 3 |
| TLR2 | Toll Like Receptor 2 |
