# Supplementary material for: Integrated Bioinformatics and Validation Reveal IL1B and Its Related Molecules as Potential Biomarkers in Chronic Spontaneous Urticaria
Source: Front Immunol. 2022 Mar 18;13:850993. doi: 10.3389/fimmu.2022.850993 (PMC8975268; doi:10.3389/fimmu.2022.850993)
Supplement: Supplementary Table S3 — 1033 DEmRNAs and 173 DElncRNAs of CSU [file Table_3.docx]

| mRNA | LncRNA |
| --- | --- |
| NDST2 | XLOC_000695 |
| SHC2 | XLOC_000153 |
| LOC283731 | XLOC_000495 |
| A2M | XLOC_001035 |
| CA2 | XLOC_001699 |
| GCGR | XLOC_003181 |
| HK2 | XLOC_004956 |
| HSD11B2 | XLOC_004384 |
| ICAM1 | XLOC_004576 |
| NCF1 | XLOC_005737 |
| PNP | XLOC_005849 |
| PLA2G2A | XLOC_005244 |
| SAA1 | XLOC_005587 |
| THBD | XLOC_005877 |
| TIMP3 | XLOC_006258 |
| XDH | XLOC_006994 |
| BLMH | XLOC_007020 |
| HSD17B1 | XLOC_007884 |
| KRT10 | XLOC_007438 |
| KRT2 | XLOC_008666 |
| LOR | XLOC_008781 |
| PECAM1 | XLOC_008828 |
| SELE | XLOC_008559 |
| VWF | XLOC_009152 |
| IL10 | XLOC_009382 |
| IL1B | XLOC_009249 |
| TNF | XLOC_009764 |
| IL6 | XLOC_009911 |
| BDKRB2 | TMEM116 |
| CXCR1 | XLOC_010377 |
| DRD4 | XLOC_010376 |
| PTGDS | XLOC_010855 |
| PTGS2 | XLOC_011134 |
| NCCRP1 | XLOC_011136 |
| CCL3L3 | XLOC_010856 |
| KRTAP5-5 | XLOC_011430 |
| PTRH1 | XLOC_012169 |
| KCTD11 | XLOC_012294 |
| KRTAP5-2 | XLOC_012586 |
| GPR179 | XLOC_012991 |
| EBF3 | XLOC_013368 |
| C2CD4B | lincROR |
| FAT3 | XLOC_l2_000727 |
| MSL1 | XLOC_l2_000735 |
| CENPP | XLOC_l2_001972 |
| KRTAP5-7 | XLOC_l2_001597 |
| KRTAP5-4 | XLOC_l2_002761 |
| LRRC26 | XLOC_l2_004385 |
| SPRR2G | XLOC_l2_004611 |
| FLG2 | XLOC_l2_004640 |
| SPRR2F | XLOC_l2_004771 |
| SPRR2B | XLOC_l2_004854 |
| FCGR1B | XLOC_l2_006101 |
| C1orf68 | XLOC_l2_006665 |
| KPRP | XLOC_l2_007834 |
| REP15 | XLOC_l2_007876 |
| AIM1L | XLOC_l2_008381 |
| ATF3 | LOC440792 |
| VPS37D | XLOC_l2_010511 |
| FTO | XLOC_l2_010751 |
| PEAR1 | XLOC_l2_011043 |
| BICC1 | XLOC_l2_013594 |
| DHRS4L1 | XLOC_l2_013751 |
| SERPINA3 | XLOC_l2_014048 |
| PSAPL1 | XLOC_l2_014294 |
| AADAC | XLOC_l2_014694 |
| ACACB | XLOC_l2_015585 |
| ACR | XLOC_l2_015760 |
| C12orf51 | XLOC_l2_015907 |
| RPTN | MT1L |
| KRTAP2-1 | psiTPTE22 |
| ADM | TUG1 |
| LCE6A | AOC4 |
| C17orf96 | LOC400927 |
| NEURL1B | SPRR2C |
| TTC28 | UBE2Q2P1 |
| KRTAP4-9 | C7orf40 |
| FAM25A | LOC441204 |
| RNF222 | LINC00340 |
| LRRD1 | LOC153684 |
| ANKRD36 | PMS2P4 |
| GOLGA6L10 | PAR-SN |
| KRTAP9-1 | DTX2P1-UPK3BP1-PMS2P11 |
| LOC100507055 | LOC650368 |
| PRR9 | FLJ45340 |
| LOC643669 | LOC728323 |
| LOC100505679 | LOC731779 |
| CDSN | LOC100190986 |
| CCR1 | LINC00087 |
| CST6 | C22orf34 |
| DNASE1L2 | LINC00265 |
| EMP1 | NKAPP1 |
| EN1 | MIR143HG |
| EREG | LOC100499466 |
| FABP5 | LOC283788 |
| FRZB | LOC643719 |
| GFRA3 | KRT18P55 |
| CXCL1 | LOC731275 |
| HAS1 | LOC645638 |
| HSPB2 | C1orf220 |
| IGFBP7 | CTSL1P8 |
| CYR61 | CTSL1P2 |
| AQP5 | KRT42P |
| AREG | LOC344887 |
| ARL4D | LOC387895 |
| ATP12A | LOC100288069 |
| CFB | LOC643650 |
| C5AR1 | FLJ27354 |
| CD1A | LOC100132352 |
| CD69 | LOC100131564 |
| CLC | LOC100132356 |
| CTPS | SRGAP2P2 |
| CTSL1 | LOC400958 |
| DES | KRT19P2 |
| DPP4 | MGC39584 |
| HBEGF | LOC728978 |
| EGR1 | LOC285084 |
| FCN1 | LOC100505839 |
| FLG | LOC100287765 |
| FPR1 | LOC729506 |
| FPR3 | LOC644554 |
| GARS | LOC100505648 |
| GPX2 | LOC100505495 |
| CXCL2 | LOC100506124 |
| HAL | XLOC_001257 |
| HDC | H19 |
| HMGB1 | PYY2 |
| HMOX1 | MIAT |
| HOXB2 | GUSBP2 |
| HOXB3 | LOC100170939 |
| HOXB5 | GUSBP11 |
| HSPA4 | DMTF1 |
| IRF1 | C17orf69 |
| ISG20 | LINC00173 |
| JUNB | LOC100133050 |
| KRT15 | PMS2P5 |
| KRT19 | FTX |
| KRT33B | SMA4 |
| KRT83 | LOC653075 |
| KRT86 | SNHG3 |
| LRP4 | LOC100507547 |
| MMP12 | LOC254128 |
| MNDA | LOC100506930 |
| MSMB | LOC100505702 |
| MYC | LOC100506990 |
| NFIX | XLOC_002996 |
| PI3 | LOC729668 |
| PIP | GUSBP1 |
| MAPK4 | GOLGA8A |
| MAPK13 | XLOC_001339 |
| PTX3 | XLOC_002997 |
| RGS1 | XLOC_004525 |
| RGS16 | XLOC_004598 |
| S100A3 | XLOC_004924 |
| S100A7 | XLOC_005005 |
| S100A8 | XLOC_005327 |
| S100A9 | XLOC_005341 |
| SERPINB4 | XLOC_006721 |
| CCL2 | XLOC_009191 |
| CCL3 | XLOC_009378 |
| CCL4 | XLOC_009509 |
| CCL18 | XLOC_013868 |
| SECTM1 | XLOC_l2_005350 |
| SLAMF1 | XLOC_l2_007770 |
| SLPI | XLOC_l2_009883 |
| UAP1 | XLOC_l2_010330 |
| SPRR1B | XLOC_l2_011987 |
| SQLE | XLOC_l2_012083 |
| SRM | XLOC_l2_014830 |
| TG | XLOC_l2_015752 |
| THBS1 | HOXA11-AS1 |
| TIMP1 | HOTAIR |
| VGF | SCARNA9 |
| ZFP36 |  |
| ZKSCAN1 |  |
| ANXA9 |  |
| HSD17B6 |  |
| FCGBP |  |
| IER3 |  |
| ZNF259 |  |
| SOCS3 |  |
| CH25H |  |
| BYSL |  |
| CAPN5 |  |
| FOXS1 |  |
| KRT33A |  |
| NEURL |  |
| DUSP1 |  |
| DUSP2 |  |
| DUSP5 |  |
| EPS8 |  |
| FOXD1 |  |
| OVOL1 |  |
| TSPAN7 |  |
| AXIN2 |  |
| HERC2 |  |
| NMI |  |
| DDX21 |  |
| LHX2 |  |
| GDF15 |  |
| IER2 |  |
| AQP3 |  |
| GPR183 |  |
| MARS |  |
| ADAMTS4 |  |
| GFPT2 |  |
| FGFBP1 |  |
| RND3 |  |
| BCL3 |  |
| CSTA |  |
| ETS2 |  |
| FOS |  |
| GPR20 |  |
| FFAR3 |  |
| FFAR2 |  |
| HAS2 |  |
| HAS3 |  |
| SALL2 |  |
| CCL13 |  |
| TIE1 |  |
| FOSL1 |  |
| IVL |  |
| KRT6A |  |
| KRT7 |  |
| KRT16 |  |
| LCN2 |  |
| S100A12 |  |
| CCL8 |  |
| NAMPT |  |
| PDZK1IP1 |  |
| MPHOSPH6 |  |
| CWC27 |  |
| MFAP1 |  |
| MT1A |  |
| MT1B |  |
| MT1H |  |
| MT1X |  |
| MT2A |  |
| SNAI1 |  |
| SPRR1A |  |
| SPRR2A |  |
| TBX2 |  |
| SFN |  |
| NFE2 |  |
| NNMT |  |
| PCP4 |  |
| PKN2 |  |
| PRPH |  |
| AGR2 |  |
| MCAM |  |
| RPP40 |  |
| HSPH1 |  |
| NOXA1 |  |
| NMU |  |
| FOSB |  |
| MARCO |  |
| CD86 |  |
| HOXA7 |  |
| HOXC9 |  |
| HOXD3 |  |
| SERPINB3 |  |
| SPRR2D |  |
| NPIP |  |
| TNFAIP6 |  |
| GALNT6 |  |
| GPR182 |  |
| BRD3 |  |
| CHIC2 |  |
| LMOD1 |  |
| KLK9 |  |
| STEAP1 |  |
| GNL2 |  |
| SERTAD1 |  |
| C13orf15 |  |
| PSORS1C2 |  |
| HOXD9 |  |
| LCE2B |  |
| LAMP3 |  |
| LYPD3 |  |
| HPGDS |  |
| SERHL2 |  |
| TFCP2L1 |  |
| SH3PXD2A |  |
| URB2 |  |
| ARHGEF17 |  |
| TRIM66 |  |
| KIAA0020 |  |
| PLXND1 |  |
| PRUNE2 |  |
| SLC39A14 |  |
| PANX1 |  |
| MYRIP |  |
| SOSTDC1 |  |
| KIF26A |  |
| ZNF593 |  |
| RRP15 |  |
| CRNN |  |
| SRRM2 |  |
| NOP16 |  |
| PLEK2 |  |
| SYT17 |  |
| NRN1 |  |
| HOXC10 |  |
| FERMT1 |  |
| THG1L |  |
| PAK1IP1 |  |
| TMEM45A |  |
| DOK5 |  |
| LRRC59 |  |
| YOD1 |  |
| ZNF395 |  |
| MAML3 |  |
| IL20 |  |
| ERRFI1 |  |
| UTS2R |  |
| CRCT1 |  |
| HOXA5 |  |
| SERTAD4 |  |
| IL36G |  |
| PNO1 |  |
| STOX2 |  |
| AKR1B10 |  |
| CXCR7 |  |
| C12orf5 |  |
| SLURP1 |  |
| ASPHD2 |  |
| OSM |  |
| STARD9 |  |
| KIAA1462 |  |
| VPS18 |  |
| SH2B2 |  |
| AQP9 |  |
| HPR |  |
| MST1 |  |
| KRT34 |  |
| KRTAP5-8 |  |
| HAMP |  |
| WFDC1 |  |
| ZNF462 |  |
| HAPLN2 |  |
| PGM5 |  |
| RNF123 |  |
| POPDC2 |  |
| NOD2 |  |
| TNS1 |  |
| HOXC8 |  |
| ELOVL4 |  |
| AEN |  |
| FNDC4 |  |
| LYNX1 |  |
| EBF1 |  |
| C1orf35 |  |
| PRR15L |  |
| LRRC2 |  |
| C1orf115 |  |
| RERGL |  |
| TTC21B |  |
| MMRN2 |  |
| NANOG |  |
| DENND2D |  |
| ZC3H12A |  |
| CENPT |  |
| SPSB1 |  |
| MYCT1 |  |
| KIAA0226L |  |
| ANKRD36B |  |
| TRIB1 |  |
| TET1 |  |
| C17orf28 |  |
| SAA2 |  |
| VMP1 |  |
| KRTAP1-3 |  |
| KRTAP1-1 |  |
| KRTAP9-9 |  |
| KRTAP4-6 |  |
| EPPK1 |  |
| PLVAP |  |
| CRISPLD2 |  |
| KRTAP4-12 |  |
| KRTAP1-5 |  |
| KRTAP3-1 |  |
| KRTAP3-2 |  |
| KRTAP4-8 |  |
| KRTAP9-3 |  |
| KRTAP9-8 |  |
| KRTAP17-1 |  |
| CCDC8 |  |
| C2orf40 |  |
| C15orf48 |  |
| CNFN |  |
| KRTAP4-4 |  |
| LCE3D |  |
| ZNF503 |  |
| C13orf33 |  |
| CSRNP1 |  |
| KRTAP2-2 |  |
| KRTAP4-1 |  |
| KRTAP4-7 |  |
| KRTAP4-2 |  |
| NKD2 |  |
| KRTAP2-4 |  |
| KRTAP3-3 |  |
| KRTAP4-3 |  |
| KRTAP4-5 |  |
| KRTAP9-4 |  |
| UCN2 |  |
| BOC |  |
| FOXQ1 |  |
| CNTNAP3 |  |
| ZNF486 |  |
| CYYR1 |  |
| LRG1 |  |
| RBM33 |  |
| HTRA3 |  |
| TWIST2 |  |
| MUCL1 |  |
| C20orf160 |  |
| SRXN1 |  |
| WFDC12 |  |
| TSPAN18 |  |
| ACER1 |  |
| SPACA4 |  |
| AHNAK2 |  |
| CTHRC1 |  |
| TMEM45B |  |
| BTBD16 |  |
| PRRT2 |  |
| LRRC25 |  |
| GYLTL1B |  |
| RDH12 |  |
| ZNF362 |  |
| HOXA9 |  |
| ASPRV1 |  |
| PYDC1 |  |
| NPHP3 |  |
| MAMDC2 |  |
| SPRR4 |  |
| KRT6C |  |
| NR4A3 |  |
| GTF2IRD2 |  |
| HOXC12 |  |
| FAAH2 |  |
| C19orf59 |  |
| BPIFC |  |
| CBX7 |  |
| LYG2 |  |
| C7orf74 |  |
| ZADH2 |  |
| S100A7A |  |
| MT1M |  |
| GSDMA |  |
| GPIHBP1 |  |
| LCE1A |  |
| LCE1B |  |
| LCE1C |  |
| LCE1E |  |
| LCE4A |  |
| LCE2A |  |
| LCE2C |  |
| LCE2D |  |
| LCE3B |  |
| LCE3E |  |
| LCE5A |  |
| MARCH3 |  |
| C1QTNF9 |  |
| CD300E |  |
| KRTAP19-1 |  |
| KRTAP19-2 |  |
| KRTAP19-5 |  |
| KRTAP20-2 |  |
| MFSD4 |  |
| KRTAP12-2 |  |
| KRTAP12-1 |  |
| TMEM119 |  |
| OLFML2A |  |
| THEM5 |  |
| GOLGA6L9 |  |
| EMB |  |
| KANK3 |  |
| VSTM1 |  |
| CLEC4G |  |
| VWA2 |  |
| ENHO |  |
| ZC3H6 |  |
| KRTAP10-7 |  |
| C9orf169 |  |
| FBXL22 |  |
| KRTAP26-1 |  |
| LYPD2 |  |
| C10orf99 |  |
| KRTDAP |  |
| LOC100132439 |  |
| SNORA12 |  |
| XLOC_001892 |  |
| XLOC_l2_005020 |  |
| XLOC_l2_012388 |  |
| XLOC_l2_011118 |  |
| XLOC_013853 |  |
| LOC729468 |  |
| XLOC_007052 |  |
| XLOC_004187 |  |
| XLOC_013994 |  |
| XLOC_l2_006021 |  |
| XLOC_l2_014098 |  |
| XLOC_l2_001399 |  |
| EMR3 |  |
| EGFL7 |  |
| CHRM1 |  |
| CKM |  |
| ATP1A2 |  |
| ATP1B2 |  |
| SMPD3 |  |
| SPRR2E |  |
| GCH1 |  |
| MYOC |  |
| TGM1 |  |
| TNNT2 |  |
| CYBB |  |
| EGR2 |  |
| MAT1A |  |
| NCF2 |  |
| LDLR |  |
| PMS2 |  |
| CCR5 |  |
| IGFBP2 |  |
| IGF2 |  |
| IGF1 |  |
| SELL |  |
| CSF3 |  |
| GSTM2 |  |
| ITGAX |  |
| PTGER2 |  |
| PTGS1 |  |
| TAGLN |  |
| HN1 |  |
| RAB11FIP1 |  |
| METRNL |  |
| HIST2H3A |  |
| EDA |  |
| LRRC37A2 |  |
| CHAC2 |  |
| ARSI |  |
| CCDC66 |  |
| KLK6 |  |
| LGR6 |  |
| ACTBL2 |  |
| CD34 |  |
| CHI3L2 |  |
| OBFC2A |  |
| MYO19 |  |
| CXCL12 |  |
| HIST2H4B |  |
| DMKN |  |
| CMTM5 |  |
| ARL17B |  |
| NBPF10 |  |
| GPER |  |
| CD53 |  |
| SPP1 |  |
| LFNG |  |
| TGFB1I1 |  |
| METTL12 |  |
| SLA |  |
| TUBB2A |  |
| TSC2 |  |
| TTC39A |  |
| LIPK |  |
| AKR1B15 |  |
| FAM162B |  |
| HRH1 |  |
| GGT5 |  |
| LIPN |  |
| SH2D5 |  |
| CCR2 |  |
| METTL21A |  |
| ZNF562 |  |
| CLDN5 |  |
| CIZ1 |  |
| ALOX12B |  |
| DOK3 |  |
| SH3BP2 |  |
| MYOCD |  |
| LIMS2 |  |
| SH3D21 |  |
| DDHD2 |  |
| STEAP1B |  |
| BIRC3 |  |
| SBSN |  |
| SUN1 |  |
| SPEG |  |
| CD14 |  |
| ZBED6 |  |
| RGNEF |  |
| COG4 |  |
| LEPR |  |
| STEAP4 |  |
| CASQ2 |  |
| RCAN2 |  |
| LDB2 |  |
| CTNND2 |  |
| CTSL2 |  |
| FPR2 |  |
| CXCR2 |  |
| ALOX5AP |  |
| POLR3D |  |
| CD33 |  |
| CCR3 |  |
| EDN1 |  |
| GBP1 |  |
| GLRX |  |
| GNA15 |  |
| GNAZ |  |
| HCK |  |
| INHBB |  |
| MEF2C |  |
| MMP1 |  |
| MMP3 |  |
| MMP7 |  |
| MMP19 |  |
| MRC1 |  |
| TNFRSF11B |  |
| PDE9A |  |
| PIM1 |  |
| PYGB |  |
| RAP1GAP |  |
| RGS2 |  |
| SNCG |  |
| TCF4 |  |
| TEAD4 |  |
| TGM3 |  |
| TYROBP |  |
| PTP4A1 |  |
| HIST1H2AM |  |
| HIST2H2AC |  |
| HIST1H3B |  |
| HIST1H4D |  |
| HIST1H4H |  |
| HIST1H4E |  |
| RGS5 |  |
| ITGA8 |  |
| LY6D |  |
| AOC3 |  |
| MATN4 |  |
| DOK2 |  |
| OSMR |  |
| BCL2A1 |  |
| CSRP1 |  |
| EMX2 |  |
| GNG11 |  |
| GZMB |  |
| CD83 |  |
| CD163 |  |
| FAP |  |
| VIPR1 |  |
| VNN2 |  |
| SPARCL1 |  |
| AIF1 |  |
| ITM2A |  |
| APOBEC3B |  |
| DEFB4A |  |
| DSC1 |  |
| KLK7 |  |
| HP |  |
| CEBPD |  |
| MAP3K8 |  |
| HIST1H1D |  |
| HIST1H1E |  |
| METTL1 |  |
| NFIL3 |  |
| NUP98 |  |
| PTPN14 |  |
| SLC20A1 |  |
| GABBR2 |  |
| LAMA5 |  |
| LMO2 |  |
| SMO |  |
| KLF10 |  |
| PPIF |  |
| MPZL2 |  |
| TUBA4A |  |
| RAN |  |
| IFI30 |  |
| DDX17 |  |
| NOP56 |  |
| BATF |  |
| WNT6 |  |
| CGREF1 |  |
| HPSE |  |
| MAP3K4 |  |
| EBNA1BP2 |  |
| SLC2A3 |  |
| ADAMTS1 |  |
| ADAMTS8 |  |
| SLC6A14 |  |
| FOXI1 |  |
| IL36RN |  |
| MAFF |  |
| SPDEF |  |
| KLK5 |  |
| RPA4 |  |
| GREM1 |  |
| TMEM2 |  |
| RAB26 |  |
| GNL3 |  |
| LHX6 |  |
| IL37 |  |
| SRPX2 |  |
| OTX1 |  |
| PPP6R1 |  |
| SPEN |  |
| SMG1 |  |
| ABI3BP |  |
| POT1 |  |
| KANK2 |  |
| SAMD4A |  |
| GADD45B |  |
| PLA2G3 |  |
| CD207 |  |
| ETV7 |  |
| ASB2 |  |
| RBM19 |  |
| CPA4 |  |
| ISYNA1 |  |
| HIGD1B |  |
| TBX3 |  |
| ARL17A |  |
| TNFRSF12A |  |
| ZAK |  |
| SOX6 |  |
| LYAR |  |
| TESC |  |
| TPCN1 |  |
| ZFP64 |  |
| RIOK2 |  |
| GIMAP5 |  |
| TREM1 |  |
| DEFB103B |  |
| BATF3 |  |
| ELMOD1 |  |
| HOXC5 |  |
| SNTG2 |  |
| UNC93A |  |
| ROBO4 |  |
| AVEN |  |
| RETN |  |
| JPH2 |  |
| PCDH19 |  |
| SORBS2 |  |
| ZNF273 |  |
| CD209 |  |
| IL22RA1 |  |
| CARD18 |  |
| TCL1A |  |
| SAMSN1 |  |
| RBM15 |  |
| NDRG4 |  |
| C3orf52 |  |
| CLMN |  |
| TMC5 |  |
| EPHX3 |  |
| TRMT2B |  |
| POF1B |  |
| TRIM45 |  |
| KIAA1683 |  |
| LY6G6C |  |
| WNT3 |  |
| PPP1R14C |  |
| ADAMTS10 |  |
| TCF7L1 |  |
| TEX101 |  |
| KREMEN1 |  |
| SLITRK6 |  |
| ING5 |  |
| ECE2 |  |
| LLPH |  |
| ZBED3 |  |
| TUBA1C |  |
| CCL14 |  |
| SLC46A2 |  |
| RHPN2 |  |
| TNKS1BP1 |  |
| TRIM10 |  |
| GNG7 |  |
| PIK3IP1 |  |
| OSR2 |  |
| MYLK |  |
| SDCBP2 |  |
| TBX1 |  |
| DPH3P1 |  |
| ASGR2 |  |
| MRVI1 |  |
| GIMAP1 |  |
| LRRC15 |  |
| SYNPO2 |  |
| SMTN |  |
| TLR4 |  |
| NRXN2 |  |
| CRB3 |  |
| RAET1E |  |
| ADAD2 |  |
| ADCY4 |  |
| LGI4 |  |
| KLK8 |  |
| CISH |  |
| MAP2K3 |  |
| PIDD |  |
| SDR9C7 |  |
| FAM69B |  |
| FGD5 |  |
| PRICKLE1 |  |
| GIMAP7 |  |
| HOXA3 |  |
| HOXC6 |  |
| CSF3R |  |
| RGS20 |  |
| PLEKHH2 |  |
| IL4I1 |  |
| FRMD3 |  |
| CLEC14A |  |
| HIST2H2AB |  |
| XG |  |
| DNMT3A |  |
| SMOX |  |
| CCRL1 |  |
| FAM83C |  |
| ZDHHC21 |  |
| NCOA7 |  |
| KRT80 |  |
| LYPD5 |  |
| GPATCH4 |  |
| PSG8 |  |
| GRHL3 |  |
| PDPN |  |
| PTGER3 |  |
| LRRC37A3 |  |
| MED8 |  |
| USP33 |  |
| MUSTN1 |  |
| RAPH1 |  |
| TPM2 |  |
| LOC100133190 |  |
| PYGM |  |
| DEFA3 |  |
| ITPR2 |  |
| WFIKKN2 |  |
| SERPINA1 |  |
| SERPINE1 |  |
| SULT2B1 |  |
| SERPINE2 |  |
| FAS |  |
| CDKN1C |  |
| SLC5A1 |  |
| C1QB |  |
| CD55 |  |
| ITGAM |  |
| ADRA1B |  |
| ADRA2A |  |
| ADRA2C |  |
| ABCB1 |  |
| PLAUR |  |
| HSP90AA1 |  |
| AHDC1 |  |
| UGT1A6 |  |
| PDE5A |  |
| DLG1 |  |
| ACPP |  |
| ACTA1 |  |
| SLC25A36 |  |
| SLC14A1 |  |
| NBPF15 |  |
| PRDM1 |  |
| HLA-DQB2 |  |
| ARG1 |  |
| CNN1 |  |
| FLNC |  |
| DNAJA1 |  |
| DARC |  |
| ITGA7 |  |
| MAL |  |
| SCGB2A1 |  |
| SCGB2A2 |  |
| PPP1R12B |  |
| P2RX1 |  |
| PDE2A |  |
| SERPINB5 |  |
| PRSS2 |  |
| TCEB3 |  |
| TNNI2 |  |
| PHLDA2 |  |
| KCNK5 |  |
| TNFSF14 |  |
| TNFRSF10C |  |
| EIF2S2 |  |
| KCNK6 |  |
| CDH6 |  |
| KCNB1 |  |
| KCNJ8 |  |
| MMP9 |  |
| SCO2 |  |
| BCAT1 |  |
| IFI16 |  |
| NR2F1 |  |
| MLL |  |
| AFF1 |  |
| NR4A2 |  |
| SCGB1D2 |  |
| SCGB1D1 |  |
| SPINK5 |  |
| ABCA8 |  |
| LILRA4 |  |
| SERPINB13 |  |
| CLEC2D |  |
| CELSR1 |  |
| SLC7A11 |  |
| SLC39A2 |  |
| DNTTIP2 |  |
| PLEKHM1 |  |
| SYNE2 |  |
| MTHFD1L |  |
| OGG1 |  |
| CLIC5 |  |
| FAM118A |  |
| CYCS |  |
| TMPRSS4 |  |
| HLA-DQA2 |  |
| CTNNBIP1 |  |
| SLC5A6 |  |
| SLC8A1 |  |
| MCL1 |  |
| PRSS22 |  |
| HIF3A |  |
| LILRA6 |  |
| GIMAP6 |  |
| EFHD1 |  |
| SERPINB1 |  |
| COL21A1 |  |
| PLEKHN1 |  |
| LAT2 |  |
| PPP1R14A |  |
| FAM125B |  |
| SLC26A9 |  |
| CDKN1A |  |
| DACT3 |  |
| APOBEC3A |  |
| SYNM |  |
| TNFRSF25 |  |
| GJB4 |  |
| SLC22A7 |  |
| PRSS36 |  |
| TUBB8 |  |
| PKIG |  |
| PLCB4 |  |
| PLEKHG5 |  |
| DPH3 |  |
| XKRX |  |
| DACT2 |  |
| NUAK1 |  |
| ACTA2 |  |
| SCN4B |  |
| ACTG2 |  |
| SMARCA4 |  |
| FCGR3A |  |
| GUCY1A3 |  |
| SLC25A25 |  |
| NTRK3 |  |
| SOD2 |  |
| MYH11 |  |
| SERPINB7 |  |
| CYP4F2 |  |
| FCGR2A |  |
| HLA-C |  |
| CAV1 |  |
| P2RX7 |  |
| PRSS3 |  |
| PTPRB |  |
| PYGL |  |
| INPP4B |  |
| GJC1 |  |
| KCNK7 |  |
| TNK2 |  |
| LILRB2 |  |
| MTHFD2 |  |
| LILRB4 |  |
| LILRB3 |  |
| DNAJC2 |  |
| ST6GALNAC1 |  |
| NR2F2 |  |
| MS4A7 |  |
| GJB3 |  |
| RERG |  |
| KCNJ15 |  |
| ABCA12 |  |
| CYP4F22 |  |
| MYL9 |  |
| CYP2C18 |  |
| CYP4F3 |  |
| CYP4B1 |  |
| TLK2 |  |
| CNTNAP3B |  |
| NLRC3 |  |
| THSD4 |  |
| IL20RA |  |
| ZNF90 |  |
| TLE4 |  |
| CLEC2A |  |
| HIST1H2AC |  |
| TCF7L2 |  |
| SLC2A14 |  |
| FAM160B1 |  |
| RPS4X |  |
| MPZL3 |  |
| MKL2 |  |
| MCOLN3 |  |
| MACROD2 |  |
| LRRC7 |  |
| PPA2 |  |
| PLIN2 |  |
| RAPGEF3 |  |
| CEP68 |  |
| DENND3 |  |
| DIP2C |  |
| DOCK9 |  |
| FRMD4A |  |
| HIP1 |  |
| HIP1R |  |
| HMBOX1 |  |
| HOXA6 |  |
| HSPA8 |  |
| JAM2 |  |
| KRTAP9-6 |  |
| KRTAP9-7 |  |
| SOX13 |  |
| SP9 |  |
