# Supplementary material for: Integrated Bioinformatics and Validation Reveal IL1B and Its Related Molecules as Potential Biomarkers in Chronic Spontaneous Urticaria
Source: Front Immunol. 2022 Mar 18;13:850993. doi: 10.3389/fimmu.2022.850993 (PMC8975268; doi:10.3389/fimmu.2022.850993)
Supplement: Supplementary Table S5 — The GO and KEGG enrichment of DEPRGs [file Table_5.docx]

The top ten lists of function enrichment analyses of DEPRGs

| Category | Description | Count | Genes |
| --- | --- | --- | --- |
| GO BP | GO:0001817 regulation of cytokine production | 10 | CD14,SERPINB1,GBP1,HMGB1,IFI16,IL1B,IRF1,P2RX7  TNF,PANX1 |
| GO BP | GO:0032611 interleukin-1 beta production | 6 | SERPINB1,HMGB1,IFI16,P2RX7,TNF,PANX1 |
| GO BP | GO:0032651 regulation of interleukin-1 beta production | 6 | SERPINB1,HMGB1,IFI16,P2RX7,TNF,PANX1 |
| GO BP | GO:0032612 interleukin-1 production | 6 | SERPINB1,HMGB1,IFI16,P2RX7,TNF,PANX1 |
| GO BP | GO:0032652 regulation of interleukin-1 production | 6 | SERPINB1,HMGB1,IFI16,P2RX7,TNF,PANX1 |
| GO BP | GO:0001819 positive regulation of cytokine production | 8 | CD14,HMGB1,IFI16,IL1B,IRF1,P2RX7,TNF,PANX1 |
| GO BP | GO:0032731 positive regulation of interleukin-1 beta production | 5 | HMGB1,IFI16,P2RX7,TNF,PANX1 |
| GO BP | GO:0032732 positive regulation of interleukin-1 production | 5 | HMGB1,IFI16,P2RX7,TNF,PANX1 |
| GO BP | GO:0038061 NIK/NF-kappaB signaling | 5 | BIRC3,CD14,HMGB1,IL1B,TNF |
| GO BP | GO:0032757 positive regulation of interleukin-8 production | 4 | CD14,HMGB1,IL1B,TNF |
| GO CC | GO:0045121 membrane raft | 4 | BIRC3,CD14,PECAM1,TNF |
| GO CC | GO:0098857 membrane microdomain | 4 | BIRC3,CD14,PECAM1,TNF |
| GO CC | GO:0000323 lytic vacuole | 3 | SERPINB1,GZMB,IL1B |
| GO CC | GO:0005764 lysosome | 3 | SERPINB1,GZMB,IL1B |
| GO MF | GO:0001530 lipopolysaccharide binding | 3 | CD14,HMGB1,P2RX7 |
| GO MF | GO:0005125 cytokine activity | 4 | HMGB1,IL1B,TNF,IL36G |
| GO MF | GO:0048018 receptor ligand activity | 4 | HMGB1,IL1B,TNF,IL36G |
| GO MF | GO:0030546 signaling receptor activator activity | 4 | HMGB1,IL1B,TNF,IL36G |
| GO MF | GO:0030545 signaling receptor regulator activity | 4 | HMGB1,IL1B,TNF,IL36G |
| GO MF | GO:0005126 cytokine receptor binding | 3 | IL1B,TNF,IL36G |
| KEGG pathway | hsa04621 NOD-like receptor signaling pathway | 7 | BIRC3,GBP1,IFI16,IL1B,P2RX7,TNF,PANX1 |
| KEGG pathway | hsa05133 Pertussis | 4 | CD14,IL1B,IRF1,TNF |
| KEGG pathway | hsa04064 NF-kappa B signaling pathway | 4 | BIRC3,CD14,IL1B,TNF |
| KEGG pathway | hsa05146 Amoebiasis | 4 | CD14,SERPINB1,IL1B,TNF |
| KEGG pathway | hsa04668 TNF signaling pathway | 4 | BIRC3,IL1B,IRF1,TNF |
| KEGG pathway | hsa05332 Graft-versus-host disease | 3 | GZMB,IL1B,TNF |
| KEGG pathway | hsa04940 Type I diabetes mellitus | 3 | GZMB,IL1B,TNF |
| KEGG pathway | hsa05144 Malaria | 3 | IL1B,PECAM1,TNF |
| KEGG pathway | hsa05134 Legionellosis | 3 | CD14,IL1B,TNF |
| KEGG pathway | hsa04640 Hematopoietic cell lineage | 3 | CD14,IL1B,TNF |
