# Supplementary material for: Integrated Bioinformatics and Validation Reveal IL1B and Its Related Molecules as Potential Biomarkers in Chronic Spontaneous Urticaria
Source: Front Immunol. 2022 Mar 18;13:850993. doi: 10.3389/fimmu.2022.850993 (PMC8975268; doi:10.3389/fimmu.2022.850993)
Supplement: Supplementary Table S7 — Hub genes-mirRNAs [file Table_7.docx]

| Genes MiRNAs | |
| --- | --- |
| HMGB1 | hsa-miR-17-5p,hsa-miR-23a-3p,hsa-let-7b-5p,hsa-miR-23b-3p  hsa-miR-143-3p,hsa-miR-193b-3p,hsa-miR-151a-3p  hsa-miR-193a-3p,hsa-miR-34a-5phsa-miR-33a-3p |
|  |  |
| IL1B | hsa-miR-587,hsa-miR-204-5p,hsa-miR-21-5p |
| IRF1 | hsa-miR-17-5p,hsa-miR-92b-3p,hsa-miR-4284,hsa-miR-9-5p  hsa-miR-374a-5p,hsa-miR-497-5p,hsa-miR-363-3p,hsa-miR-24-3p  hsa-miR-449a,hsa-miR-548ak,hsa-miR-548c-5p,hsa-miR-32-5p  hsa-miR-92a-3p,hsa-miR-548i,hsa-miR-4690-5p,hsa-miR-20b-5p  hsa-miR-548am-5p,hsa-miR-106b-5p,hsa-miR-15a-5p,hsa-miR-26b-5p  hsa-miR-20a-5p,hsa-miR-106a-5p,hsa-miR-301a-3p,hsa-miR-141-5p  hsa-miR-449b-5p,hsa-miR-548w,hsa-miR-212-3p,hsa-miR-548d-5p  hsa-miR-33b-3p,hsa-miR-195-5p,hsa-miR-424-5p,hsa-miR-548au-5p  hsa-miR-15b-5p,hsa-miR-1292-5p,hsa-miR-4446-3p,hsa-miR-103a-3p  hsa-miR-301b-3p,hsa-miR-548y,hsa-miR-335-5p,hsa-miR-26a-5p  hsa-miR-548j-5p,hsa-miR-93-5p,hsa-miR-130b-3p,hsa-miR-454-3p  hsa-miR-107,hsa-miR-25-3p,hsa-miR-548o-5p,hsa-miR-367-3p  hsa-miR-4437,hsa-miR-625-5p,hsa-miR-130a-3p,hsa-miR-16-5p |
|  |  |
| P2RX7 | hsa-miR-125a-5p,hsa-miR-570-3p,hsa-miR-20a-5p,hsa-miR-216b-5p  hsa-miR-588,hsa-miR-10b-5p,hsa-miR-1275 |
| TNF | hsa-miR-130a-3p |

The mirRNAs targeting the hub genes was predicted via theTargetScan, miRNet and DIANA TOOLS TarBase v.8 databases
