# Supplementary material for: Integrated Bioinformatics and Validation Reveal IL1B and Its Related Molecules as Potential Biomarkers in Chronic Spontaneous Urticaria
Source: Front Immunol. 2022 Mar 18;13:850993. doi: 10.3389/fimmu.2022.850993 (PMC8975268; doi:10.3389/fimmu.2022.850993)
Supplement: Supplementary Table S8 — MiRNAs-lncRNAs [file Table_8.docx]

| MiRNAs | LncRNAs |
| --- | --- |
| hsa-mir-17-5p | HOTAIR, H19 |
| hsa-mir-34a-5p | TUG1 |
| hsa-mir-151a-3p | TUG1,SCARNA9 |
| hsa-mir-23a-3p | LINC00173 |
| hsa-mir-23b-3p | LINC00173 |
| hsa-let-7b-5p | LINC00265 |
| hsa-mir-193a-3p | H19 |
| hsa-mir-193b-3p | H19 |
| hsa-mir-21-5p | SNHG3 |
| hsa-mir-20a-5p | HOTAIR, H19 |
| hsa-mir-216b-5p | TUG1, H19, FTX |
| hsa-mir-588 | TUG1, GUSBP11, H19 |
| hsa-mir-125a-5p | GUSBP11 |

The lncRNA targeting the miRNA was predicted via miRNet and got the intersection with the DElncRNAs
