# Supplementary material for: Integrated Bioinformatics and Validation Reveal IL1B and Its Related Molecules as Potential Biomarkers in Chronic Spontaneous Urticaria
Source: Front Immunol. 2022 Mar 18;13:850993. doi: 10.3389/fimmu.2022.850993 (PMC8975268; doi:10.3389/fimmu.2022.850993)
Supplement: Supplementary file 8 [file Table_2.docx]

| Group | Name | Age | Gender | Disease course |
| --- | --- | --- | --- | --- |
| CSU | Siy*** Han | 6 | M | 2 years |
| CSU | M** Gong | 39 | W | 6 months |
| CSU | Lany*** Wang | 59 | W | 4 months |
| CSU | Xiuf** Qiu | 37 | W | 3 months |
| CSU | Z**** Long | 48 | M | 2 months |
| CSU | S*** Liu | 13 | W | 2 years |
| CSU | Y*** Li | 43 | W | 2 months |
| CSU | Mingl**** Song | 27 | M | 2 months |
| CSU | Zhir** Zhou | 2 | M | 2 months |
| CSU | Hans***** Fan | 14 | W | 2 months |
| Control | Y*** Tang | 32 | W | / |
| Control | Chengl** Li | 48 | M | / |
| Control | Canh** He | 31 | W | / |
| Control | J** Jiang | 36 | W | / |
| Control | Y** Zhang | 24 | W | / |
| Control | W*** He | 42 | M | / |
| Control | Ler*** Li | 26 | W | / |
| Control | Y*** Wang | 39 | M | / |
| Control | H*** Zhang | 40 | W | / |
| Control | E* Wang | 35 | M | / |
